# Logic minimization and rule extraction for identification of functional sites in molecular sequences

**DOI:** 10.1186/1756-0381-5-10

**Published:** 2012-08-16

**Authors:** Raul Cruz-Cano, Mei-Ling Ting Lee, Ming-Ying Leung

**Affiliations:** 1 Department of Epidemiology and Biostatistics, University of Maryland, College Park, MD, USA; 2 Department of Mathematical Sciences, Bioinformatics Program, Border Biomedical Research Center, The University of Texas at El Paso, El Paso, TX, USA

## Abstract

**Background:**

Logic minimization is the application of algebraic axioms to a binary dataset with the purpose of reducing the number of digital variables and/or rules needed to express it. Although logic minimization techniques have been applied to bioinformatics datasets before, they have not been used in classification and rule discovery problems. In this paper, we propose a method based on logic minimization to extract predictive rules for two bioinformatics problems involving the identification of functional sites in molecular sequences: transcription factor binding sites (TFBS) in DNA and O-glycosylation sites in proteins. TFBS are important in various developmental processes and glycosylation is a posttranslational modification critical to protein functions.

**Methods:**

In the present study, we first transformed the original biological dataset into a suitable binary form. Logic minimization was then applied to generate sets of simple rules to describe the transformed dataset. These rules were used to predict TFBS and O-glycosylation sites. The TFBS dataset is obtained from the TRANSFAC database, while the glycosylation dataset was compiled using information from OGLYCBASE and the Swiss-Prot Database.

We performed the same predictions using two standard classification techniques, Artificial Neural Networks (ANN) and Support Vector Machines (SVM), and used their sensitivities and positive predictive values as benchmarks for the performance of our proposed algorithm. SVM were also used to reduce the number of variables included in the logic minimization approach.

**Results:**

For both TFBS and O-glycosylation sites, the prediction performance of the proposed logic minimization method was generally comparable and, in some cases, superior to the standard ANN and SVM classification methods with the advantage of providing intelligible rules to describe the datasets. In TFBS prediction, logic minimization produced a very small set of simple rules. In glycosylation site prediction, the rules produced were also interpretable and the most popular rules generated appeared to correlate well with recently reported hydrophilic/hydrophobic enhancement values of amino acids around possible O-glycosylation sites. Experiments with Self-Organizing Neural Networks corroborate the practical worth of the logic minimization method for these case studies.

**Conclusions:**

The proposed logic minimization algorithm provides sets of rules that can be used to predict TFBS and O-glycosylation sites with sensitivity and positive predictive value comparable to those from ANN and SVM. Moreover, the logic minimization method has the additional capability of generating interpretable rules that allow biological scientists to correlate the predictions with other experimental results and to form new hypotheses for further investigation. Additional experiments with alternative rule-extraction techniques demonstrate that the logic minimization method is able to produce accurate rules from datasets with large numbers of variables and limited numbers of positive examples.

## Background

### Logic minimization techniques

Logic minimization is the application of algebraic axioms to a binary function in order to reduce the number of digital variables and/or rules needed to express the function; the technique is also known as logic simplification or reduction. In other words, the purpose of logic minimization is to find a simplified representation for a given function requiring the minimum number of logic operations and variables for its implementation. In designing a digital system, if an equivalent function of the system can be achieved with fewer components, the design will increase its reliability and decrease the manufacturing cost. Mano [1] provides an excellent introduction to these techniques.

The starting point for logic minimization techniques is the desired functionality, which can be summarized in some algorithmic forms by logic equations or in the form of a table with the desired input/output patterns as in the present study. This table is known as a function table. Since logic minimization axioms can only be applied to binary patterns, the input/output patterns must be encoded as binary strings before being subjected to any logic minimization procedure.

Each binary pattern (string or example) in the dataset can also be interpreted as a digital logic rule stating the correct output for a given set of values of the input variables. Using logic minimization, a long set of rules can be reduced to a set using fewer variables and/or rules to perform the same function. In other words, for any pattern of values for the input variables, both the original and reduced sets of rules produce the same output, i.e., they are equivalent.

For example, the 16 input patterns of Table 1 can be reduced to the rules shown in Table 2, in which variable *A* has been discarded from the rules because it is completely irrelevant in determining the values of the output. Both tables have the same interpretation, if (*B* = 0 and *D* = 0) or (*C* = 1) then the output is 1, while in any other cases, the output is 0. The 0-output rule is implicit in Table 2. In an input pattern, the symbol “-” means that the value of a particular input variable is irrelevant to determine the output. In an output pattern, we used “-” to denote that the output for a given input pattern is unknown.

**Table 1:** Binary Function before Logic Minimization

**Pattern**	**Inputs**				**Output**
	**A**	**B**	**C**	**D**	
1	0	0	0	0	1
2	0	0	0	1	0
3	0	0	1	0	1
4	0	0	1	1	1
5	0	1	0	0	0
6	0	1	0	1	0
7	0	1	1	0	-
8	0	1	1	1	-
9	1	0	0	0	1
10	1	0	0	1	0
11	1	0	1	0	1
12	1	0	1	1	1
13	1	1	0	0	0
14	1	1	0	1	0
15	1	1	1	0	-
16	1	1	1	1	-

**Table 2:** Binary Function after Logic Minimization

**Pattern/Rule**	**Inputs**				**Output**
	**A**	**B**	**C**	**D**	
1	-	0	-	0	1
2	-	-	1	-	1

In Table 2, it should be noted that rule number 2 would assign an output of 1 to the patterns with unknown output in Table 1 (patterns 7, 8, 15, and 16). This output value was not randomly assigned; instead, the logic minimization technique assigned to the unknown outputs the numeric value (0 or 1) that produced the simplest set of equivalent rules. In other words, if the outputs of patterns 7, 8, 15, or 16 were assumed to be 0, the length of Table 2 would increase. This capability of assigning an output value to a previously unseen input pattern is the basis of our classification and prediction system. These patterns are referred to as test set. It is a situation similar to an ANN dealing with an unseen input pattern. In theory, the output could be any value in the range of the output variable but the ANN generalizes the information obtained from the training set, i.e. the input patterns with known output, and takes an educated guess about what the most likely value for this output would be.

It is practically impossible to find the optimal solution manually, even for relatively small problems with a few dozens of input variables; hence, it is desirable to find algorithms which can reduce the function table in a systematic manner. This characteristic would make the algorithms suitable to be transformed into computer programs. The first of these techniques to become widely accepted was the Quine- McCluskey method developed in 1956 [2].

The main idea behind this method is the iterative application of the axiom that for any binary variable *A*, *A + Ā =*1, where *Ā* stands for the negation of *A,* and the symbol + represents the operation “or”. For any two binary variables *X* and *Y*, the result of an *X + Y* operation is 1 if *X* or *Y* is equal to 1. Notice that in the case *A* is equal to 1 then *A + Ā = 1 + 0 =* 1. If *A* is equal to 0 then *Ā =* 1 and therefore *A + Ā =* 0 + 1 = 1*.* To appreciate how this principle can be applied to reduce expressions, just follow the next example:

(1) $$\bar{A\;}BC+ABC=1\to \left(\bar{A}+A\right)\;BC=1\to \left(1\right)\;BC=1\to BC=1$$

Notice that we were able to find a reduced expression which will produce the same output as the original rule for any pattern of input values.

Using letters to represent the variables in all the expressions is not the most efficient way to do it in a computer. It is better to a designate a position in a function table for each variable and then to represent the values of the variable with 0 or 1. For example, if the first position of the expression corresponds to the variable *A*, the second to *B,* and the third to *C*, then the previous example can be written as seen in Table 3.

**Table 3:** Binary Function represented in a table before minimization

**Pattern**	**Inputs**			**Output**
	**A**	**B**	**C**	
1	1	1	1	1
2	0	1	1	1

After the minimization, the table would look like Table 4.

**Table 4:** Binary Function represented in a table after minimization

**Pattern**	**Inputs**			**Output**
	**A**	**B**	**C**	
1	-	1	1	1

In other words, variable *A* can now be ignored because is not relevant for predicting the output. Using the binary equation representation, it is easy to design a computer program to identify the possible reductions in a dataset by looking for patterns that differ from each other in just one position, i.e., the values are the same except for one variable. In our example, this variable is *A*. Partially reduced patterns can be combined with other reduced patterns until they cannot be simplified anymore (“-” can be interpreted as 0 or 1, depending on what is more convenient for the logic minimization algorithm).

Although the Quine-McCluskey algorithm can be relatively easy to implement, its application requires doubling the amount of memory and computing time every time a new variable is added, hence the resources needed to apply it grow exponentially with the number of input variables. As a result, the Quine-McCluskey method is practical only for applications with a small number of input variables.

A different approach is implemented by the ESPRESSO algorithm [3]. This algorithm relies on multidimensional representations of the function table which allow the conglomeration of similar input patterns with equal outputs into a smaller set of more general patterns (i.e., patterns including “-”). In general, ESPRESSO requires less memory with around 16% less computation time than Quine-McCluskey [4]. While the Quine-McCluskey and ESPRESSO algorithms are different, the mathematical principles behind both algorithms are the same. A more detailed explanation can be found in [5]. The input for ESPRESSO is a function table of the desired input–output patterns; the result is a table with an approximation of the minimum number of input/output patterns required to perform the function described in the input table. This method does not guarantee the global minimum, but in practice, has been found to be very close to it most of the time.

While logic minimization techniques have been applied to inferring gene regulatory networks from gene expression patterns [6-9], they have not been much used for other bioinformatics problems, such as classification and rule discovery in molecular sequences analysis. In this paper, we apply the method to predict TFBS in DNA and then to the identification of O-glycosylation sites in mucin-type proteins.

TFBS are short segments of DNA near the transcription initiation site where transcription factors bind to regulate the transcription process. Though the binding sites for a given transcription factor are generally composed of similar sequences of nucleotides, there can be considerable variability. We will use logic minimization techniques to predict the TFBS for the transcription factor SOX9, which is a member of the SOX protein family that play critical roles in the regulation of numerous developmental processes, including sex determination, skeleton formation, pre-B and T-cell development and neural induction [10].

Glycosylation is an important posttranslational modification for more than half of all the proteins in a cell. O-glycosylation refers to the event in which a carbohydrate is covalently linked to the hydroxyl group of serine (S) or threonine (T) residues, and is one of the three main types of posttranslational modifications involving carbohydrates. This modification influences a number of properties of proteins including proteolytic resistance, solubility, immunological properties, ligand binding and is also involved in recognition during sperm-egg binding. O-glycosylation site prediction remains as a difficult problem because no amino acid consensus sequence is known [11].

## Methods

### Method based on logic minimization

The number of binary variables generated during the encoding stage might be too large to be handled by any digital logic minimization software without exhausting the physical resources of advanced computers. Even programs implementing ESPRESSO have difficulties handling more than a dozen of binary variables, for example the software used in this research has a limit of 22 input variables [3]. This limitation requires the use of a technique for selecting the most relevant features in a dataset. Also, by keeping only this relatively small number of variables for the rule extraction, it is easier to observe all of them at once and, hence, to discover patterns or trends. A similar argument has been suggested in [12].

**Figure 1:**
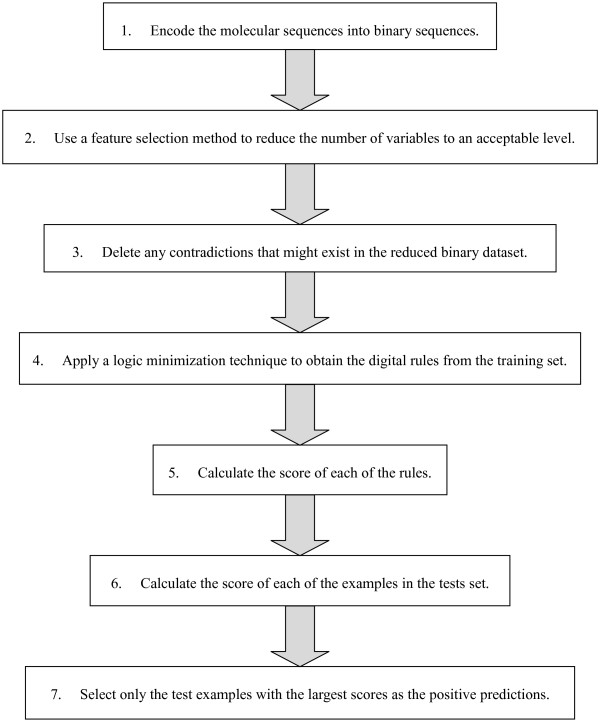
Logic Minimization-Based Algorithm.

If more than 22 variables are deemed significant, the essence of the algorithm described in Figure 1 can still be applied unchanged. Its implementation would have to be adapted to the dataset being studied, for example, different logic minimization software (more powerful than ESPRESSO) might be required.

More variables would require more time and resources, so the user would have to find a point of balance between the number of variables to be included in the experiments and the extra time and effort needed to process them. The point of balance will depend on each user. A user with a high-performance computer can use many more variables than a user with a standard desktop who needs to find the rules quickly. Each individual user of the algorithm will have to decide on the compromise that works best. Preliminary studies with few variables can lead to accurate estimations of the time and resources needed for larger versions of the problem at hand.

The technique selected to perform this feature selection was Support Vector Machines (SVM). Details about how SVM can help to select the top variables are presented later in this paper. The main reason for the selection of SVM is its dual nature as classifier/feature selector, a similar characteristic shared by our proposed method which is a classifier/ rule extractor. Other reasons are: 1) there is software available; 2) they are a well-respected and popular technique; 3) they are also fast and computationally inexpensive; and 4) they provide deterministic and therefore consistent results.

Another issue when using logic minimization techniques is that it requires encoding all the available information in a digital manner; depending on the scheme used this could produce contradictions in the dataset. For example, as shown in Table 5, the input data are in floating point format, so we need to transform it to binary. If we decide to set each value equal to 1 if it is greater than .5, then we would get the data in Table 6.

**Table 5:** Input/Output Patterns

**Pattern**	**Inputs**		**Output**
	**A**	**B**	
1	.8	.9	1
2	.7	.8	0

**Table 6:** Input/Output Patterns

**Pattern**	**Inputs**		**Output**
	**A**	**B**	
1	1	1	1
2	1	1	0

Notice that we have a contradiction in Table 6 because the same pattern of input values produces different outputs. The axioms of digital logic would not work in this case, so it is imperative to find an adequate encoding system in avoiding contradictions or to erase them from the dataset before applying a logic minimization algorithm. In the example shown in Tables 5 and 6, moving the threshold for being considered a positive value from .5 to .7 would be enough to solve the contradiction. The software used in both cases presented in this report includes functions designed to produce binary datasets free of contradictions.

In theory, it is easy to build an example where the threshold cannot be found. Suppose that we have a variable *X* with values 0, 0.5 and 1, and assume that the classifications for these examples are 0, 1, and 0, respectively. In this case, there is no threshold dividing the classes based on variable *X*. Real-life applications usually have many variables, providing a much better chance to separate the classes. Even if the threshold cannot be found, the user will gain a better understanding of the dataset. Knowing that the variable *X* does not have direct relationship with the desired output, the user can then try different transformations of the data to solve the problem. For example, if instead of *X*, we use *f*(*X*) = |*X* -.5| then f(0) = .5 (class = 0), f(.5) = 0 (class = 1) and f(1) = .5 (class = 0), now a threshold of .25 would separate the classes just fine. A different alternative would be to separate the variable in more than two partitions using more than one threshold. For example, we can use three binary variables to represent if *X* < .25, .25 < = *X* < = .75, and *X* > 75.

Another problem with the existing logic minimization methods is that the number of times that each rule/pattern appears in the dataset is lost. For example, both rules in Table 2 appear four times in the original dataset. Rule 1 of Table 2 is observed in patterns 1, 3, 9, and 11 of Table 1, while rule 2 applies to patterns 3, 4, 11 and 12.

It is conceivable that some of the rules in the reduced set represent larger numbers of patterns from the original dataset than others. In other words, these rules are more “popular”. It is also possible for a single input pattern to fit several rules in the reduced set simultaneously. If a previously unseen input pattern is compared against the reduced set of rules, it would be assigned a simple output of 0 or 1, depending on which rule(s) it fits. For the positive predictions (output = 1), there would not be any way to determine which of these test patterns comply with rules that represent a large number of input patterns in the original dataset or if they fit several rules at the same time. If a previously unseen input pattern is predicted positive (output = 1) by more popular rules, or by multiple rules, it would be reasonable to consider it as more likely to be a true positive.

We therefore decided to add another layer to the logic minimization method. First, we count the times that each rule appears in the training set and call this number the score of the rule. Then, for each input pattern *i* in the test set, we add the scores of the rules the pattern satisfies. This number is considered the score of test example *i*. Finally, instead of recording as a positive prediction, all the patterns that satisfy at least one rule, only the test examples with the top scores can be picked as positive predictions. This allows increasing the positive predicting value (PPV) of the method, i.e., the percentage of positive predictions turned out to be correct. The trade-off for this higher PPV is a decrease in the sensitivity, i.e., the percentage of positive examples that are correctly identified. By having this scoring mechanism to adjust the balance between sensitivity and PPV, the proposed method can be adapted to work efficiently in different situations to fit the desired level of sensitivity and PPV in the specific application. For example, one would want higher sensitivity when dealing with cancer detection, but higher PPV for non-critical medical conditions that are expensive to verify.

We summarize the main steps of the algorithm based on logic minimization in Figure 1. Similar to the standardization or the use of logarithmic scale that might or might not affect the results of some experiments for a given classifier, the transformation of the datasets to a binary representation might or might not affect the results of the proposed algorithm for a given dataset. Alternative techniques can help to confirm that the results provided by the logic minimization algorithm were not affected by the discretization of the data.

### Support vector machines

SVM feature selection methods were studied first in 2002 [13] and have been used in various biological applications such as prediction of single-nucleotide polymorphisms [14], location of hot-spot residues at protein-protein interfaces [15], finding small non-coding RNA in bacterial genomes [16], monitoring of blood glucose [17], and gene selection in DNA microarray data analysis [18]. A detailed description of the specific type of SVM used in this paper can be found in [19].

The modification of the parameters of the SVM in order to get the desired results is known as training. Usually, the behavior desired for an SVM is obtained by providing it with examples of inputs and the corresponding observed outputs. This set of patterns is referred to as the training set.

For SVM, the classification of the examples is performed by a decision function

(2) $$D\left(X\right)=W^{T}G\left(X\right)+b$$

where *G* is vector of non-linear functions of size *L*, where *L > > n*, actually *L* might be infinite. The elements of the vector *W = (w*_*1*_*,w*_*2*_*,…,w*_*L*_*)* and the bias term *b* are real value numbers.

The generalization ability of an algorithm is defined as its classification performance for the unknown data. The generalization abilities of the decision function can be maximized by increasing the margin, i.e., the distance between a decision function and the input vector nearest to it. The hard-margin support vector machines are decision functions giving the maximum margin. Hard-margin SVMs may not always exist in real-life problems. One can get around this situation by introducing slack terms; the resulting systems are known as soft-margin SVM. It is also necessary to find the values of the parameters (support vectors) for the non-linear functions. For this project, it has been decided to consider all the patterns in the dataset as support vectors.

The change in the generalization capability, i.e. the change in the margin, of a SVM created by erasing a variable can be accurately estimated [20]. The process of repetitively eliminating from the data set the variable which produces the smallest change in the generalization capability of a SVM is called recursive feature elimination (RFE) [13]. To obtain information about the programs corresponding to the SVM-RFE method consult [21].

The dual nature of SVM, which can be both classifiers and feature selectors, allows us to address two different goals. First they help our algorithm by reducing the number of variables that we need to include during the logic minimization stage. Then, after the digital rules are determined, the SVM, along with ANN, provide performance benchmarks to compare with the logic minimization approach. Although their mathematical frameworks are similar, different software packages are better suited for the two goals. We use “SVM and Kernel Methods Matlab Toolbox” [21] for feature selection and “LS-SVMlab Toolbox” [22] for performance comparison purposes.

### Artificial neural networks (ANN)

ANN is a mathematical model based on the human brain. Its behavior depends on the strengths of the connections, also called the weights of the network, among simple processing units or neurons. The modification of the weights of the network in order to get the desired results is known as training. The training of an ANN basically consists of providing to it examples of inputs and the corresponding observed outputs. Examples in bioinformatics include prediction of promoter sites on DNA sequences [23], protein secondary structure prediction [24], and automatic classification of protein sequences [25].

The analysis in the next section is performed with feed-forward networks with five hidden neurons, initialized using the algorithm proposed in [26] and trained with the Marquart-Levenberg algorithm [27]. Detailed description of the ANN used can be found in [28].

Neural Networks trained with the Marquart-Levenberg algorithm have been successfully used in bioinformatics problems such as the data analysis and parameter determination of Protein-Lipid System [29] and the prediction of MHC Class 11-binding Peptides [30]. The Neural Network Toolbox of MATLAB was used for performance comparison against the other methods.

### Rule extraction from SVM and ANN

Although there are several methods designed to extract rules from trained SVM and ANN [31]; they suffer from many disadvantages, such as producing a large number of rules [32,33], limited explanation capability [34-36], low accuracy [37], sensibility to noise [38], requiring pre-processing of the data, which might not suitable for categorical features [39], or the comprehensibility decays with large number of examples and/or variables [40]. All of these algorithms require the application of a complex procedure to the trained SVM or ANN to extract the rules, while in the case of the logic minimization method proposed in the present study, the creation of the classification system and the discovery of the rules describing the dataset are the same, without the need to implement additional steps (see Figure 1).

Another complication is that the computer programs corresponding to these rule extraction methods are not available on the Internet. Furthermore, the great majority of their experiments are limited to problems from the University of California at Irvine (UCI) Machine Learning repository [41] with a relatively small number of variables.

The fidelity of a rule system is the percentage of test patterns where the rule system’s classification agrees with the original SVM or ANN classification. All the algorithms mentioned in this section produce rule systems with high fidelity; hence, their performance is similar to that of the original classification system from which these rules were extracted [34,35,38]. For this reason, we assume that the performance numbers shown in the next section for ANN and SVM would not improve even if these systems were translated into sets of rules.

The lack of availability of rule extraction software for SVM is a problem noticed in [42]. On the other hand, it is possible to find software for extracting rules from ANN. “Recognize, Predict, Forecast!TM” is a software using Self-Organizing Neural Networks (SONN) to solve classification and regression problems [43]. This software presents the ANN as a graph helpful for understanding what input characteristics are used by the ANN in making a prediction. Moreover, in trained SONNs, only the essential connections are drawn for making the rule extraction task possible for a scientist with a basic understanding of ANN.

One difficulty in using this software is that it requires either 50% or 75% of the data for training. In case of TFBS, it would be extremely expensive to study half or three quarters of thousands of all possible TFBS and then make predictions in the remaining candidates. Although “Recognize, Predict, Forecast!TM” is useful for validating the extracted rules, this limited testing capability makes it unsuitable for performance comparisons with the feed-forward ANN, SVM, and the proposed method. In any case, SONNs are designed to be understandable at the expense of accuracy; hence, it is reasonable to assume that their performance for more realistic training test sets would not be better than the performance of a feed-forward ANNs.

## Results and discussion

### Transcription factor binding sites

#### Transcription factor binding sites data sets

In recent years, an enormous and still increasing output of DNA sequence data and annotation information have been obtained by a series of genome sequencing projects for humans and model organisms. A key issue in modern molecular biology is to understand the mechanisms of transcriptional regulation.

The binding of transcription factors to specific DNA sequences near the transcription start site is one of the first steps determining whether a gene is turned on or off. Although the sequence variability in these TFBS makes it difficult to identify all the potential sites precisely, the well-characterized features of these TFBS make it possible to model them statistically [44]. There are techniques specifically designed to predict TFBS based on relevant motif discovery [45-47], and the logic-minimization method can be applied to any classification or prediction problem with little change.

The TFBS dataset used in this research consists of 73 experimentally verified binding sites (each with a length of 14 nucleotides) bound by the transcription factor SOX9. The data are obtained from TRANSFAC database where TFBS profiles are being maintained and updated. To make the data suitable for the logic-minimization techniques, the nucleotides in the sequences are transformed to binary values: A is represented by 1000, C by 0100, G by 0010 and T by 0001. After the transformation, each TFBS is represented by 56 binary variables (i.e., 14 nucleotides in each TFBS by four possible values for each nucleotide). Other encoding schemes have been considered but we reach the conclusion that, while these encoding schemes would lead to shorter training and test samples, the user would lose the main advantage of the proposed algorithm, i.e., its ability to provide rules that can be interpreted immediately by any scientist who might not have training in digital design, mathematics, machine learning, etc. In other words, there would not be a clear relationship between each binary variable and unique nucleotide/amino acid in a certain position. Instead, each binary variable would now represent a set of two or more of the original features that would require careful examination to be determined.

A total of 10 training sets and their corresponding test sequences were generated. Each of the training sets consisted of around 150 input/output patterns with 90% of the known TFBS and a comparable number of random human sequences that were also 14 nucleotides long. Each training set had 65 or 66 TFBS (90% of 73 known TFBS) and the other sequences were pieces of random human DNA. These training sets were used as the starting point for the creation of the three prediction systems compared in this research: SVM, ANN and the logic minimization method. Since each of the training sets contained a different subset of the 73 known TFBS and different pieces of random human DNA, each of them produced a different collection of prediction systems.

To test the efficiency of the three different prediction methods studied in this study, the remaining seven or eight (about 10% of 73) TFBS were arbitrarily inserted in a 20,000 nucleotide-long sequence of randomly chosen human DNA. A total of 10 test sequences were created, one for each training set.

Each of the nucleotides in the human background sequence was also encoded as a binary string. A sliding window of 56 binary numbers was then moved across the human DNA sequence and then each method tried to predict if the start of such window is the start of a TFBS or not. In other words, each window was interpreted as a 56-bit binary sequence with a corresponding output of 0 (i.e., not a TFBS) or 1 (i.e., a TFBS). A positive prediction within two nucleotides of the start of a TFBS was considered a correct prediction.

#### Transcription factor binding sites results

In the present study, ESPRESSO requires that all the possible binary combinations for the 22 top variables (as determined by the SVM-RFE) have an assigned output. In other words, we have to create a file with all the 2^22^ = 4,194,304 possible patterns and assigned them an output value. Notice that we have only around 150 patterns in a training set, meaning that many of the patterns required in the ESPRESSO input file are not included. Actually, once the 22 variables are selected using SVM-RFE, many training input patterns are mapped to the same reduced binary pattern. On average, the training sets are reduced to 75+/− different patterns. The rest of the possible patterns in the ESPRESSO input file are assigned an output of “-”.

For all the 10 training sets, ESPRESSO found that only the top seven variables were necessary to accurately express the logic rules defining the TFBS. These variables and their corresponding interpretation are shown in Table 7. Although some of the variables listed in Table 7 are dependent, they are not redundant. For example, variables 26 and 25 might seem redundant because both refer to position 10 but if variable 26 is 0 (i.e., “Nucleotide 7 is not C”) then variable 25 could be 0 or 1 (i.e., “Nucleotide 7 might or might not be A”). The development of a mechanism capable of incorporating known existing dependencies in this step would require the implementation of a new logic minimization method (Step #4 of Figure 1). We decide to prove that the proposed algorithm can produce a competitive performance using the well-known and recognized ESPRESSO.

**Table 7:** Variables deem necessary by the Espresso to predict TFBS

**Ranking**	**Variable**	**Meaning**
1	26	Nucleotide 7 is/is not C
2	33	Nucleotide 9 is/is not A
3	29	Nucleotide 8 is/is not A
4	40	Nucleotide 10 is/is not T
5	23	Nucleotide 6 is/is not G
6	39	Nucleotide 10 is/is not G
7	25	Nucleotide 7 is/is not A

The median number of rules required to express the training sets is only two (plus the implicit 0-output rule). Moreover, the same rules appear many times despite the random nature of the training sets; there are only seven different rules among the 20+ rules obtained from the 10 different datasets.

For example, the rules for dataset #3 are shown in Table 8. Their interpretation would be the following:

 If “Nucleotide 9 is A” and “Nucleotide 8 is A” and “Nucleotide 10 is T” and “Nucleotide 6 is G” then the sequence is a TFBS.

 If “Nucleotide 7 is C” and “Nucleotide 9 is A” and “Nucleotide 8 is A” and “Nucleotide 10 is T” and “Nucleotide 10 is not G” and “Nucleotide 7 is not A” then the sequence is a TFBS.

 Everything else is not a TFBS (Notice that this rule is not explicitly shown in Table 8).

**Table 8:** Rules for Prediction of TFBS after Logic Minimization

**Rules**	**Inputs**							**Output**
	**26**	**33**	**26**	**40**	**23**	**39**	**25**	
1	-	1	1	1	1	-	-	1
2	1	1	1	1	-	0	0	1

The rules can be further reduced if the known dependencies among the variables are integrated. For this particular training set and its corresponding test sequence, seven of the eight TFBS inserted in the 20,000 nucleotides sequence were found using these 3 simple rules. Besides the seven correct predictions the digital-logic system also predicted 219 possible starts of TFBS turned out to be false. If only the predictions with the top scores, i.e., those comply with both of the first two rules, are considered a positive prediction, the number of predicted TFBS is reduced to 130 while still capturing the same seven true TFBS. This is a fortunate coincidence as a smaller number of predictions, even if they are more accurate, usually lead to a lower sensitivity.

In general, for each test set, the corresponding digital rules generate around 250 positive predictions, which are a small fraction of the 19,986 possible places to start a complete TFBS in a test sequence. This number is reduced to around 130 if only the examples in the test set that satisfy all the rules are considered possible TFBS. Although these numbers of predictions are a small number compared to the possible starts of TFBS, it is a large number compared to the seven or eight TFBS to be found, this produces the small PPVs seen in Table 9. Both SVM and ANN suffer from similar woes. The top 250 predictions are selected for SVM and ANN in the same fashion as described in [28] and [19]. The comparison of the performance of the different systems is shown in Table 9. Notice that the digital logic-rules systems achieve a performance similar to that of the other well-known techniques. In addition, the resulting systems can be expressed in a language that can be understood by any researcher interested in studying TFBS. It is also notable that the usage of the scores for the test set can lead to doubling the PPV while reducing the sensitivity only by a small percentage (around 7%). If all the 56 binary variables are included in the training and test sets, the performance of both the ANN (sensitivity = 44.64%, PPV = 1.43%) and SVM (Sensitivity = 83.93%, PPV = 2.69%) is negatively affected compared to the results listed in Table 9. It seems that by adding that many variables for a dataset with such a small number of examples leads to overfitting of the ANN and SVM, i.e., the models are describing the random variations in the datasets instead of the underlying general principles that exists among the variables. Moreover, the time and space required for generating the training and test sets would double with each variable added to the dataset.

**Table 9:** Comparison of Performance Results for Prediction of TFBS using the Top 7 Binary Variables

**Method**	**Sensitivity**	**PPV**
Digital Logic Rules	89.30	2.46
Top Digital Logic	82.14	5.78
SVM	87.50	2.80
ANN	55.36	1.77

For the TFBS, using all the original features during training, a SONN with 3 layers selects to connect from all the variables only the inputs 26, 29, 33 and 40 with positive values. This is basically rule 2 of Table 8.

To further reduce the number of predictions, it is possible to combine the digital-minimization method predictions with those of the other classification systems (SVM and ANN) and obtain ensemble predictions. For example, only the places “strongly” predicted as TFBS by the three methods could be considered a positive prediction. The optimal way to include the proposed algorithm as part of a larger prediction system will be explored in future research.

### Glycosylation

#### Glycosylation data sets

The existing prediction techniques for O-glycosylation are based on methods comparable to our algorithm, e.g., Nearest Neighbor Algorithm [48]. In fact, both ANN [49] and SVM [50] have been previously applied to this problem. Since the material in [50] provides better performance than ANN and uses binary data similar to the one presented in this paper, SVM will be further discussed in this section.

The glycosylation data were gathered from OGLYCBASE [51] and the Swiss-Prot Database [52]. From these protein databases, the mucin-type glycoprotein entries with verified O-glycosylation sites were extracted. For each verified S and T residue, a sequence of five amino acids before and five after it was collected, generating example sequences of 11 amino acids long, like those used in the experimental studies by Gerken *et al.*[53,54]. A total of 4264 amino acid sequence patterns for the S residue and 4507 for the T residue were collected. For the S residue, 249 patterns corresponded to O-glycosylated (positive) examples while remaining 4015 patterns were not (negative). For the T residues, there were 1668 positive and 2839 negative examples.

We used 85% of the known O-glycosylated and equal number non-O-glycosylated sites to create the classification systems. The rest of the positive examples and an equal number of the remaining negative examples were used to create a balanced test set.

Notice that the validation experiments for the O-Glycosylated dataset are different from those used for TFBS. These different criterions are the best approximation to for the situations that researchers studying each of the problems would face. In the case of the O-glycosylation data, the researchers know that only S or T can be O-glycosylated, hence they can focus only on pieces of protein that have an S or T in the center. The TFBS without this limitation and a sliding window containing all the possible substrings of DNA as potential TBFS should be employed.

Each of the 11 amino acids is encoded as a 21-bit long binary sequence, leading to a total of 231 binary variables. Each bit represents one of the 20 standard residues in alphabetical order of the one-letter amino acid code or indicates the “unknown” category for incomplete sequences. Only the position corresponding to the amino acid observed is set to one, while the rest are set to zeros. For example, the sequence ADY of amino acids Alanine (A), Aspartic acid (D), Tyrosine (Y) would be encoded as: 10000000000000000000 0010000000000000000 00000000000000000010.

This encoding scheme is similar to the one presented by Li *et al.*[50] but they use 20 amino acids around the S or T residues. Although both Li *et al*. [50] and our group use binary data and SVM in the O-glycosylation problem, there are several major differences in the approaches, mainly that in [50] there is no effort to extract information from the data, i.e., there is no feature selection from their 1200 binary variables (which would be possible using SVM) nor rule extraction (which would require a separate algorithm after SVM). It is also worth noting that the inclusion of all the features would not contribute to gaining new knowledge about this biological case study. In addition, there is no distinction between S and T O-glycosylated sites, which according to experts [54] is a vital piece of information for O-glycosylation prediction systems due to the different structure for the two cases.

#### Glycosylation data results

We used the top 20 of the 231variables selected by the SVM RFE procedure as they gave a good balance between performance and the computational resources needed to run the ESPRESSO program. Moreover, preliminary experiments with SVM showed that the performances for the SVM using top 20, 30, 40 and 50 variables were similar; hence, the inclusion of more than 20 variables in the classification systems would not be essential. ESPRESSO was unable to further reduce the number of variables. Again, the number of input patterns with known output was reduced when only the top variables were used to represent them, followed by deletion of contradictions and repetitions. There were many sequences of the 2^20^ possible binary patterns not included in our dataset, again we assigned them an output of “-”. .

The Digital Rules provide sensitivity around 90% for both the S and T datasets. This sensitivity compares favourably against SVM and ANN for both S and T cases of the O-glycosylation data as seen in Table 10. It is also better than the best result of 87% using a balanced training set presented by Li et al. [50]. The row “Top 45% Digital Logic” contains the performance for the case in which only the predictions with the top 45% of the scores, calculated in the form described earlier in the subsection “Logic Minimization Techniques” in the Methods section, are considered positive predictions. As expected this brought an increase in the quality of the predictions as shown by higher PPVs. The price for this higher PPV is a decrease in sensitivity. The PPV for a balanced training set in [50] is 80.3%, which is comparable to the best PPV obtained using the Top 45% Digital Logic Rules. The 45% is not meant to be interpreted as an optimal number in any way. It just shows how the scores of the rules can be used to tune, up to a certain point, the balance of PPV and sensitivity according to the needs of the user. Users who wish higher PPV and can tolerate lower sensitivity can select a higher percentage to be considered a positive prediction, while those who wish to have a higher sensitivity can lower the score needed to be considered a positive prediction. The “Combined Performances” columns shown in Table 10 list the result of combining the performance for the T sites with the performance for the S sites based on the number of confirmed O-Glysosylated sites for each type.

**Table 10:** Comparison of Performance Results for Prediction of O-glycosylated Sites using the Top 20 Binary Variables

**Method**	**T**		**S**		**Combined Performance**	
	**Sensitivity**	**PPV**	**Sensitivity**	**PPV**	**Sensitivity**	**PPV**
Digital Logic Rules	91.23	66.60	89.40	66.82	90.99	66.11
Top 45% Digital Logic	84.76	80.61	71.70	72.97	83.06	79.62
SVM	74.47	95.48	70.26	69.52	73.92	92.11
ANN	74.04	95.05	65.65	57.95	72.95	90.11

The high accuracy with 85% sensitivity and 80.3% PPV mentioned in [50] is obtained using a leave-one-out cross validation, in other words the test set is just a tiny fraction of the dataset, approximately 0.2%, compared to the 15% that we used. A change of this proportion in the allocation of examples for the training and test sets would lead to a boost in the performance of our method; unfortunately, the leave-one-out cross validation would require the repetition of the algorithm (creation of training/test sets, their transformation to digital rules, etc.) hundreds of times. In [49], the performance of the ANN for cross-validation has been compared against the performance on the independent new dataset where the sensitivity and PPV are on average 12.35% and 12.46% lower, respectively. The leave-one-out cross validation is known to be computationally expensive; this drawback and other disadvantages are explored in [55]. Experiments using all the 231 variables in an independent test set led to combined performances comparable to those in [50] for both the SVM (sensitivity =89.12%, PPV = 76.62% ) and the ANN (sensitivity = 81.71%, PPV = 80.97%). It is important to note that the combined results obtained using all the variables are not clearly superior to the combined performances obtained using only the top 20 significant variables (see Table 10). Any increase in the sensitivity seems to be negated by a decrease in the positive predictive value and vice versa. These results underscore the usefulness of feature selection that, at least in the case studies presented in this paper, has helped to reduce the number of variables, and hence the required computational resources and time needed to create the training and test sets, without decreasing the overall performance.

All the methods struggle with the lack of positive examples in the case of the “S” dataset. Moreover, comparing the results presented in Table 10 for T and S datasets make easy to understand the importance of having a larger amount of examples to obtain a better prediction system. In Table 10, the PPV for ANN and SVM are better than those for Digital Rules for the T dataset; nevertheless, given the superior sensitivity of the proposed method for those same cases, this result does not mean that SVM or ANN offer a better performance. Users who prefer high sensitivity would be better served by the proposed method. Moreover, if the user is also interested in gaining a better understanding of the problem, the rules provided by the proposed method can be interpreted immediately, while SVM and ANN users would have to find a rule extraction method and learn to how to use it, etc.

The equivalents to Table 7, i.e. the most important variables, are shown in Tables 11 and 12. Notice that Tables 11 and 12 share 10 variables (50%) and the possible meaning of these coincidences is that the 10 common variables are always involved in the O-glycosylation process, while the rest of the variables can help to understand what makes the O-Glycosylation process different when you have an S or a T. We gain this insight almost effortlessly due to the interpretability of the rules produced by the proposed method.

**Table 11:** Top 20 Variables deemed necessary by the SVM-RFE to predict S O-glycosylated Sites

**Ranking**	**Variable**	**Meaning**
1	183	Amino Acid in Position 3 is/is not P
2	101	Amino Acid in Position −1 is/is not T
3	143	Amino Acid in Position 1 is/is not T
4	204	Amino Acid in Position 4 is/is not P
5	99	Amino Acid in Position −1 is/is not P
6	185	Amino Acid in Position 3 is/is not T
7	206	Amino Acid in Position 4 is/is not T
8	163	Amino Acid in Position 2 is/is not S
9	88	Amino Acid in Position −1 is/is not D
10	36	Amino Acid in Position −4 is/is not P
11	180	Amino Acid in Position 3 is/is not K
12	100	Amino Acid in Position −1 is/is not S
13	2	Amino Acid in Position −5 is/is not R
14	162	Amino Acid in Position 2 is/is not P
15	227	Amino Acid in Position 5 is/is not T
16	78	Amino Acid in Position −2 is/is not P
17	142	Amino Acid in Position 1 is/is not S
18	164	Amino Acid in Position 2 is/is not T
19	74	Amino Acid in Position −2 is/is not L
20	148	Amino Acid in Position 2 is/is not A

**Table 12:** Top 20 Variables deemed necessary by the SVM-RFE to predict T O-glycosylated Sites

**Ranking**	**Variable**	**Meaning**
1	15	Amino Acid in Position −5 is/is not P
2	206	Amino Acid in Position 4 is/is not T
3	164	Amino Acid in Position 2 is/is not T
4	38	Amino Acid in Position −4 is/is not T
5	227	Amino Acid in Position 5 is/is not T
6	183	Amino Acid in Position 3 is/is not P
7	143	Amino Acid in Position 1 is/is not T
8	185	Amino Acid in Position 3 is/is not T
9	101	Amino Acid in Position −1 is/is not T
10	99	Amino Acid in Position −1 is/is not P
11	80	Amino Acid in Position −2 is/is not T
12	17	Amino Acid in Position 5 is/is not T
13	225	Amino Acid in Position −4 is/is not A
14	22	Amino Acid in Position −1 is/is not A
15	85	Amino Acid in Position 2 is/is not P
16	162	Amino Acid in Position −3 is/is not P
17	57	Amino Acid in Position 3 is/is not P
18	142	Amino Acid in Position 1 is/is not S
19	59	Amino Acid in Position −3 is/is not T
20	169	Amino Acid in Position 3 is/is not A

Each of the 15 S training sets produced around seven rules, while the 15 T training sets produced around 66 rules each. When all the positive T examples are put together with an equal number of negative examples in a single training set, the resulting digital logic system has 100 rules. A similar arrangement for the S dataset leads to 60 rules.

The interpretation of the rules that produced the performances is shown in Table 10 in the section of Additional files 1 and 2. Since not all the variables are necessary in all the rules, it is possible to provide the interpretation of the most popular rules for the S and T overall datasets in Tables 13 and 14, respectively. Those input patterns not classified as O-glycosylated by any of the discovered rules are assigned a 0 output and considered as not O-glycosylated.

**Table 13:** Most popular rule for S O-glycosylated sites

**If amino acid in position**	**is**	**Enhancement value**
−1	T	>1
1	not S	<1
2	A	>1
4	T	=1
5	not T	=1
then we have found an O-Glycosylated S		

**Table 14:** Most popular rule for T O-glycosylated sites

**If amino acid in position**	**is**	**Enhancement value**
−4	T	=1
−2	T	>1
1	Not T	<1
2	T	>1
3	Not T	<1
4	T	>1
5	Not T	<1
then we have found an O-Glycosylated T		

The number of positive sites that follow the most popular rules shown in Tables 13 and 14 are 32 positive O-glycosylated S sites and 495 positive O-glycosylated T sites, respectively. It should be noted that the most popular rules for both S and T correspond respectively to the hydrophobic and hydrophilic enhancement values for the ppGalNAc T5 transferase, a key enzyme in O-glycosylation. These enhancement values are determined experimentally for each amino acid at each position from −5 to +5 around the S or T sites by [54]. A value greater than 1 indicates that the amino acid is favored at that position while a value less than 1 suggests the contrary. For comparison with our results, we look up the enhancement value of the amino acid in the particular position as described in the two most popular rules and indicate in the rightmost column of Tables 13 and 14 whether the value is greater than, less than, or equal to 1. In each case, when our rule says that a position is a particular amino acid, the enhancement value is greater than or equal to 1. When our rule says that a position is not a particular amino acid, the enhancement value is less than or equal to 1.

The SONN was unable to handle the S O-glycosylated dataset is perhaps due to the large number of variables for such a small number of positive examples. Despite using half of the dataset with all the variables for training, its sensitivity was only around 45%. Adding layers to the SONN did not improve its performance. For this reason, we decided not to pay attention to the rules produced by the SONN for this dataset. The results for the T O-glycosylated dataset were more encouraging with a sensitivity of around 72% and PPV of 77% using half of the dataset as training set. A SONN with 4-layer selected inputs 15, 38, 99,101, 143, 164, 206, and 227, and all of these variables appear in Table 12. Even better, the architecture of the network shows that input 38, 143, 164, and 227 are associated before being conglomerated with the rest of the relevant inputs. This set of variables strongly resembles most of the highest ranked variables listed in Table 14.

Future research efforts for the O-glycosylation process should include the investigation of the correspondence between digital-rule predictions and new experimental data. Although the results mentioned in [54] already seem to confirm the veracity of the most popular rules, there are still several others with enough prominence to warrant a more meticulous examination in wet-lab experiments.

Another line of research will be the extension of the digital rules to fuzzy logic rules. Fuzzy logic is a multi-valued logic that allows representing the subjective values of truth encounter in many real-life problems. For example, there are biologists who believe that protein residues should be classified into several levels in O-glycosylation, not simply as O-glycosylated or not. This second line of research will allow us to combine the structure and order provided by the digital rules with the knowledge of one of these scientists.

## Conclusions

The proposed logic minimization method provides sets of rules for predicting TFBS on human DNA and O-glycosylation sites on proteins with sensitivity and PPV comparable to those from ANN and SVM. Furthermore, the logic minimization method has the additional capability of generating interpretable rules that allow biological scientists to correlate the predictions with other experimental results and form new hypothesis for further investigation. In addition, our investigations into alternative rule extraction methods have also demonstrated that the logic minimization method is able to produce accurate rules even from datasets with large number of variables and limited numbers of positive examples, which is a situation often encountered in biological data mining.

## Competing interests

The authors declare no competing interests.

## Authors’ contributions

RCC conceived of the algorithm proposed in the manuscript and wrote the computer programs implementing the algorithm. He also helped to draft the manuscript. MYL supervised the gathering of the data used during the research presented in the paper. Both MYL and MLTL collaborated with the writing of the manuscript and provided ideas about how to improve the algorithm and prove its value to solve real-life problems. All three authors read and approved the final manuscript.

## Supplementary Material

Additional file 1 Rules for Prediction of S O-glycosylated sites after Logic Minimization. Click here for file

Additional file 2 Rules for Prediction of T O-glycosylated sites after Logic Minimization. Click here for file
